# Anterior direct decompression significantly relieves spinal cord high signal in patients with ossification of the posterior longitudinal ligament: a case-control study

**DOI:** 10.1186/s13018-023-04388-y

**Published:** 2023-11-24

**Authors:** Zichuan Wu, Zifan Zhang, Aochen Xu, Shihao Lu, Cheng Cui, Baifeng Sun, Yang Liu

**Affiliations:** Department of Spine Surgery, Shanghai Changzheng Hospital, Naval Medical University, 200003 Shanghai, People’s Republic of China

**Keywords:** Ossification of the posterior longitudinal ligament, Cervical spondylotic myelopathy, High cord signals, Magnetic resonance imaging, Signal change ratio, Anterior approaches, Posterior approaches

## Abstract

**Background:**

In patients with cervical spondylotic myelopathy caused by ossification of the posterior longitudinal ligament, high cord signal (HCS) is frequently observed. However, limited research has investigated the variations in HCS improvement resulting from different surgical approaches. This study aims to explore the potential relationship between the choice of surgical approach and the postoperative improvement of intramedullary high signal in ossification of the posterior longitudinal ligament (OPLL) patients.

**Methods:**

We extensively reviewed the patients' medical records, based on which demographic information such as gender, age, and body mass index (BMI) were recorded, and assessed the severity of the patients' neurological status preoperatively and postoperatively by using the Japanese Orthopedic Association score (JOAs), focusing on consecutive preoperative and postoperative Magnetic resonance imaging (MRI) T2WI measurements, to study the statistical correlation between the improvement of HCS and the choice of surgical approach.

**Results:**

There were no significant differences in demographic, imaging parameters, and clinical symptoms between patients undergoing anterior and posterior surgery (*p* > 0.05, Table 1). However, both improvement in JOAs (Recovery2) and improvement in HCS (CR2) were significantly better in the anterior surgery group two years after surgery (*p* < 0.05, Table 1). Multifactorial logistic regression analysis revealed that posterior surgery and higher preoperative signal change ratio (SCR) were identified as risk factors for poor HCS improvement at the two-year postoperative period (*p* < 0.05, Table 2).

**Table 1:** Differences in demographic, imaging parameters, and clinical symptoms in patients with anterior and posterior approach

	Anterior approach	Posterior approach	P-Values
Demographic data			
Sex (male/female)	10/12	6/17	0.175
Age	58.59 ± 5.68	61.43 ± 9.04	0.215
Hypertension	14/8	14/9	0.848
Diabetes	16/6	19/4	0.425
BMI	25.58 ± 4.72	26.95 ± 4.58	0.331
Smoking history	19/3	16/7	0.175
Preoperative measured imaging parameters			
Preoperative SCR	1.615 ± 0.369	1.668 ± 0.356	0.623
CR1	0.106 ± 0.125	0.011 ± 0.246	0.08
CNR	0.33 ± 0.073	0.368 ± 0.096	0.15
C2–7 Cobb angle	8.977 ± 10.818	13.862 ± 13.191	0.182
SVA	15.212 ± 8.024	17.46 ± 8.91	0.38
mK-line INT	3.694 ± 3.291	4.527 ± 2.227	0.323
Imaging follow-up			
6 months postoperative SCR	1.45 ± 0.44	1.63 ± 0.397	0.149
2 years postoperative SCR	1.26 ± 0.19	1.65 ± 0.35	0.000**
CR2	0.219 ± 0.14	− 0.012 ± 0.237	0.000**
Clinical symptoms			
Preoperative JOAs	10.64 ± 1.59	10.83 ± 1.47	0.679
6 months postoperative JOAs	11.82 ± 1.37	11.65 ± 1.4	0.69
2 years postoperative JOAs	14.18 ± 1.01	12.52 ± 2.06	0.001**
Recovery1	0.181 ± 0.109	0.128 ± 0.154	0.189
Recovery2	0.536 ± 0.178	0.278 ± 0.307	0.001**

^*^, statistical significance (*p* < 0.05). **, statistical significance (*p* < 0.01)

BMI = body mass index. SCR = the signal change ratio between the localized high signal and normal spinal cord signal at the C7-T1 levels. CR1 = the regression of high cord signals at 6 months postoperatively (i.e., CR1 = (Preoperative SCR—SCR at 6 months postoperatively)/ Preoperative SCR). CR2 = the regression of high cord signal at 2 years postoperatively (i.e., CR2 = (Preoperative SCR—SCR at 2 years postoperatively)/ Preoperative SCR). CNR = canal narrowing ratio. SVA = sagittal vertical axis. mK-line INT = modified K-line interval. JOAs = Japanese Orthopedic Association score. Recovery1 = degree of JOAs recovery at 6 months postoperatively (i.e., Recover1 = (JOAs at 6 months postoperatively—Preoperative JOAs)/ (17- Preoperative JOAs)). Recovery2 = degree of JOAs recovery at 2 years postoperatively (i.e., Recover2 = (JOAs at 2 years postoperatively−Preoperative JOAs)/ (17−Preoperative JOAs))

**Table 2:** Linear regression analyses for lower CR2 values

	95% CI		P value
Uni-variable analyses			
Demographic data			
Sex (male/female)	− 0.01	0.221	0.924
Age	− 0.015	0.003	0.195
Hypertension	− 0.071	0.204	0.334
Diabetes	− 0.195	0.135	0.716
BMI	− 0.375	0.422	0.905
Smoking history	− 0.249	0.077	0.295
Surgical approach	− 0.349	− 0.113	0.000^#^
Preoperative measured imaging parameters			
C2–7 Cobb angle	− 0.009	0.002	0.185
SVA	− 0.008	0.008	0.995
mK-line INT	− 0.043	0.005	0.122
Preoperative SCR	0.092	0.445	0.004^#^
CR1	0.156	0.784	0.004^#^
CNR	− 0.76	0.844	0.918
Multi-variable analyses			
Surgical approach	− 0.321	− 0.118	0.000**
Preoperative SCR	0.127	0.41	0.000**
CR1	− 0.018	0.501	0.067

^#^, variables that achieved a significance level of *p* < 0.1 in the univariate analysis

*statistical significance (*p* < 0.05). **statistical significance (*p* < 0.01)

BMI = body mass index. SCR = the signal change ratio between the localized high signal and normal spinal cord signal at the C7-T1 levels. CR1 = the regression of high cord signals at 6 months postoperatively (i.e., CR1 = (Preoperative SCR—SCR at 6 months postoperatively)/ Preoperative SCR). CR2 = the regression of high cord signal at 2 years postoperatively (i.e., CR2 = (Preoperative SCR—SCR at 2 years postoperatively)/ Preoperative SCR). CNR = canal narrowing ratio. SVA = sagittal vertical axis. mK-line INT = modified K-line interval

**Conclusions:**

For patients with OPLL-induced cervical spondylotic myelopathy and intramedullary high signal, anterior removal of the ossified posterior longitudinal ligament and direct decompression offer a greater potential for regression of intramedullary high signal. At the same time, this anterior surgical strategy improves clinical neurologic function better than indirect decompression in the posterior approach.

## Introduction

Ossification of the posterior longitudinal ligament (OPLL) is a perplexing condition characterized by the peculiar occurrence of ectopic ossification within the posterior aspect of the cervical vertebrae. This abnormality leads to a narrowing of the cervical spinal canal, resulting in the compression of the precious spinal cord and delicate nerve roots [1]. The etiology of OPLL is multifaceted, involving genetic factors, chronic strain, and metabolic abnormalities, among others. The prevalence of OPLL varies greatly across populations, with Asian ethnic groups boasting a higher incidence, typically ranging from 2 to 4% [2, 3].

When it comes to imaging patients with spinal disorders, including OPLL, magnetic resonance imaging (MRI) reigns supreme due to its noninvasive nature, high-resolution capabilities, and its knack for visualizing soft tissues with remarkable finesse. MRI not only serves to elucidate the extent of spinal cord compression and stenosis but also unveils neuropathic changes by reflecting the enigmatic variations in the intensity of nerve signals [4].

T2-weighted (T2WIs) sequences are commonly employed to assess the degree of disc degeneration and spinal cord compression. In the enigmatic realm of OPLL, a localized high signal in the spinal cord (HCS) on T2WIs of MRI is a telltale sign of neurological damage, and its perplexing high signal alteration hints at non-specific pathological changes occurring within the spinal cord. These changes may manifest as edema, inflammation, vascular ischemia, glial cell proliferation, or even myelination abnormalities, ultimately resulting in the corresponding neurological symptoms that perplex medical professionals [4–6]. Astoundingly, it has been revealed that the localized high signal in the spinal cord observed on T2WIs provides a more comprehensive assessment of changes in spinal cord neurological function when compared to T1WIs [4]. In addition, previous studies have shown that HCS signal intensity on preoperative T2WI is negatively correlated with postoperative neurologic prognosis, and that postoperative regression of HCS is closely related to recovery of neurologic dysfunction [7–10].

The surgical treatment of OPLL encompasses both anterior and posterior approaches, each accompanied by promising clinical outcomes. However, the eternal debate surrounding the choice between these surgical methods perpetuates, as researchers continue to grapple with conflicting evidence [11–16]. Existing studies tentatively suggest that anterior surgery is favored for OPLL cases featuring a high occupancy rate and posterior convexity deformities. On the other hand, posterior surgery appears to be more suitable for managing long-segment OPLLs, as it offers a greater opportunity for extensive segmental decompression [2, 17, 18]. Nevertheless, when it comes to patients' postoperative neurologic recovery rates and long-term prognoses, previous studies find themselves in a state of perpetual disagreement regarding the superior approach. These studies often evaluate patients' clinical prognoses and the pros and cons of anterior and posterior surgery by employing functional scores such as the Oswestry Disability Index (ODI) and the Japanese Orthopedic Association score (JOAs), as well as assessing the degree of compression relief through measurements such as spinal canal volume and sequential changes like the cervical kyphosis angle and sagittal vertical axis (SVA). However, a notable research gap exists regarding the exploration of the divergent changes in the degree of HCS in patients following anterior and posterior surgery for OPLL cases associated with HCS. Given the profound impact of HCS on patients' clinical function, this enigmatic change could potentially serve as a valuable reference for guiding surgical procedure selection in OPLL patients, adding an extra layer of complexity to the decision-making process.

Therefore, OPLL is a perplexing condition characterized by ectopic ossification within the posterior longitudinal ligament, causing narrowing of the cervical spinal canal and compression of the spinal cord and nerve roots. Its multifaceted etiology and varying prevalence across populations add to the intricate tapestry of this condition. The use of MRI, particularly T2WIs, provides a glimpse into the perplexing neuropathic changes and their correlation with neurological function. The choice between anterior and posterior surgery in OPLL management remains a topic of heated debate, with conflicting evidence and enigmatic factors shaping the decision-making process. The study of HCS improvement may provide an important reference for the choice of surgical approach in patients with OPLL. The aim of this study was to investigate the potential relationship between the choice of surgical approach and the improvement of postoperative intramedullary high signal in patients with OPLL and to provide an important reference for the choice of surgical approach in patients with OPLL.

## Method

We extensively reviewed the patients' medical records, based on which demographic information such as gender, age, and body mass index were recorded, and assessed the severity of the patients' neurological status preoperatively and postoperatively by using the JOAs, focusing on consecutive preoperative and postoperative MRI T2WI measurements, to study the statistical correlation between the improvement of HCS and the choice of surgical approach.

### Patient enrolment and case collection

#### Source of patients

**Fig. 1:**
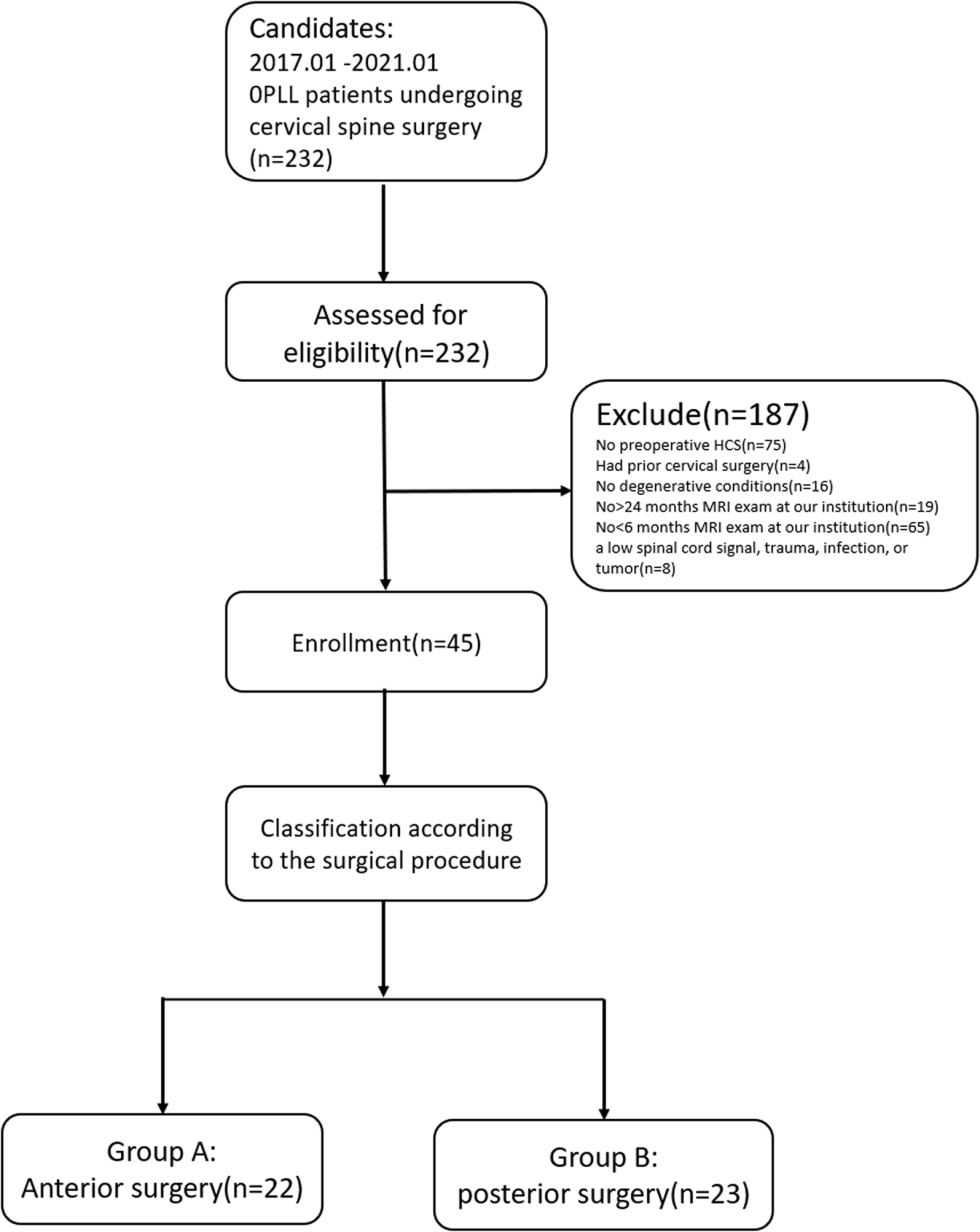
Schematic for patients inclusion and exclusion

In this retrospective clinical imaging study, OPLL patients who underwent surgery at Shanghai Changzheng Hospital's cervical spine treatment unit between December 1, 2017, and December 1, 2022 were included. We conducted preoperative and postoperative MRI examinations and followed up with these patients for approximately 24 months. Out of a total of 232 patients, 45 met the eligibility criteria for enrollment (Fig. 1).

#### Inclusion criteria

(1) Patients with ossification of the posterior longitudinal ligament of the cervical spine secondary to DCM; (2) Ossification of the posterior longitudinal ligament of the cervical spine as seen on cervical spine CT or cervical spine X-ray; (3) Compression of the spinal cord by the OPLL as seen on MRI's T2Wis resulting in increased signal intensity within the spinal cord of the corresponding segments, and an HCS (i.e., SCR ≥ 1.23) on preoperative MRI T2WIs; (4) A minimum of 24 months' follow up.

#### Exclusion criteria

Patients with prior cervical spine surgery, absence of HCS in preoperative MRI T2WIs or with low spinal cord signal, trauma, infection, or surgical treatment of tumors.

Based on the aforementioned criteria, patients were divided into two groups based on the surgical approach: anterior surgery group (*n* = 22) and posterior surgery group (*n* = 23). The anterior surgery group included anterior cervical discectomy and fusion (ACDF), anterior cervical discectomy and fusion (ACCF), and hybrid decompression and fusion (HDF), and the posterior surgery group included laminoplasty (LP), and laminectomy and fusion (LF), and all of these procedures were performed by a single senior spine surgeon. Statistical analysis revealed no significant difference (*p* > 0.05, Table 1) in the general conditions between the two groups, ensuring comparability.

### Measurement of imaging data

Core independent variables: selection of the anterior OR posterior surgical routes

Ending variables: improvement in HCS at 6 months and 2 years postoperatively (CR1 and CR2)

#### Covariate

**Fig. 2:**
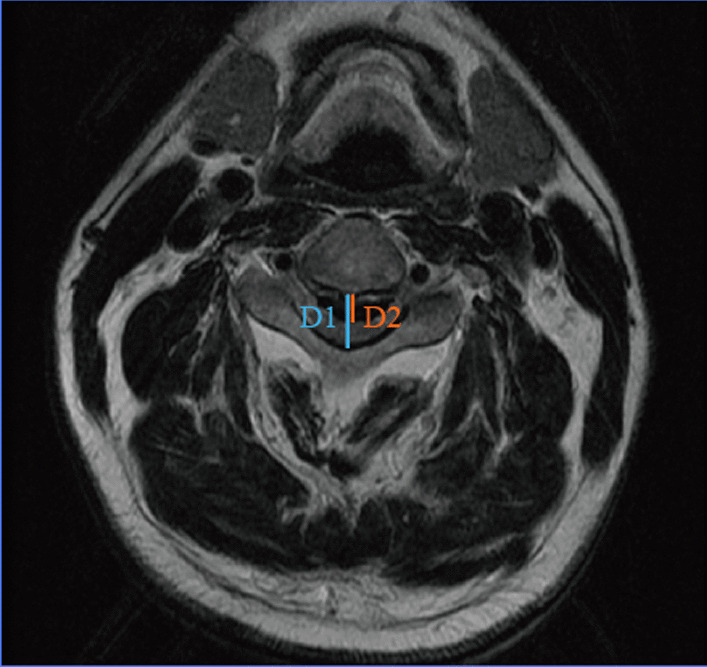
Schematic diagram for measuring CNR

*Canal narrowing ratio (CNR)* An axial image indicating CNR calculation method, CNR = D2/D1 (Fig. 2).

**Fig. 3:**
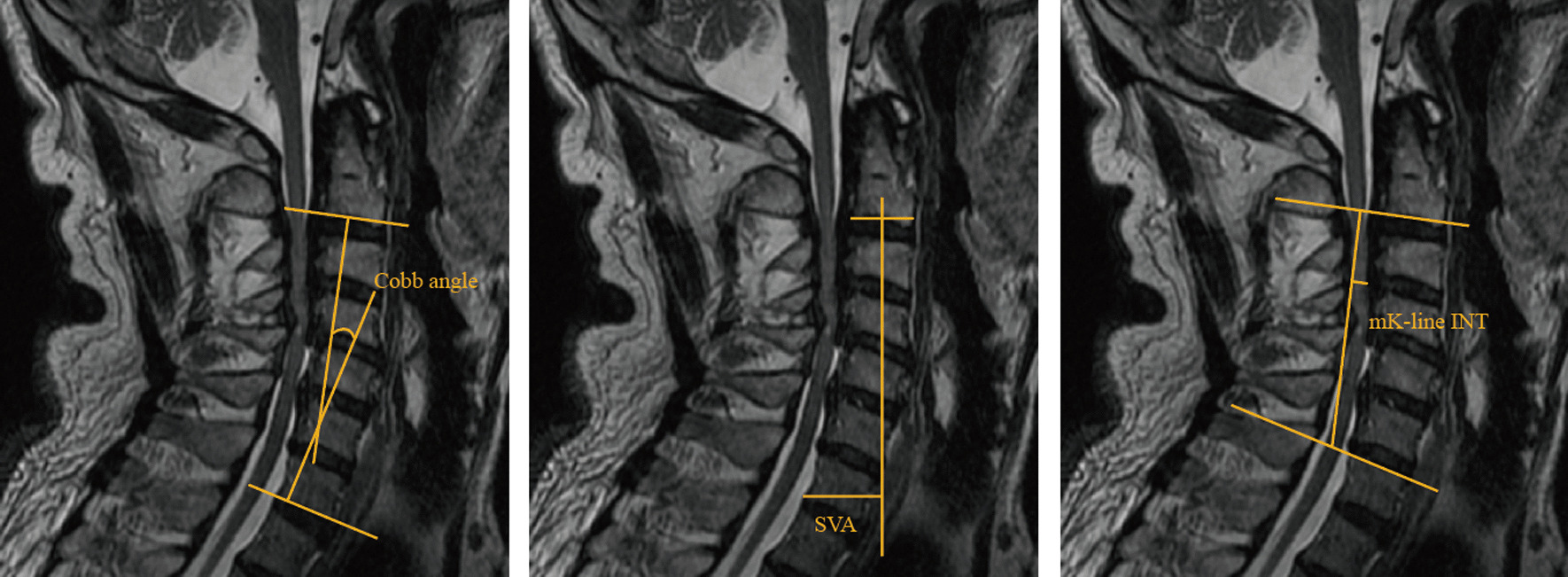
Schematic diagram of the measurement of C2–7 Cobb angle, SVA and mK-line INT

*C2–7 Cobb angle* The C2–7 Cobb angle was defined as the angle between lines drawn tangential to inferior endplates of C2 and C7 (Fig. 3).

*Sagittal vertical axis (SVA)* The C2–7 sagittal vertical axis (SVA) was defined as the horizontal distance between the vertical line from the center of the C2 vertebral body and the posterosuperior corner of the C7 vertebral body (Fig. 3).

*Modified K-line interval (mK-line INT)* mK-line INT was defined as the minimum interval between the tip of the anterior compression factor of the spinal cord and the line connecting the midpoints of the cord at the level of the inferior endplates of C2 and C7 (Fig. 3).

### Statistical analysis

SPSS 26.0 software was used to complete all the following statistical analyses *p* < 0.05 was regarded as a significant difference between groups.

To judge the interobserver and intraobserver reliability, 10 patients were randomly selected. One week after the measurement of the above-mentioned imaging parameters, values of parameters for these 10 patients were remeasured by the radiologist and a well-trained spine surgeon (who has three years of learning experience in the musculoskeletal imaging measurement group). The intraclass correlation efficiency (ICC) was computed to identify the repeatability of imaging based parameters [19–22].

As all the indicators obtained from the imaging measurements were continuous variables. All continuous variables were tested for normal distribution. Skewness and kurtosis were calculated for each continuous variable in descriptive statistics. If both were less than 1, the variable was considered to conform to a normal distribution, and vice versa, the variable was considered to be skewed. Continuous variables that conformed to normal distribution were described using mean ± standard deviation, and variables that did not conform to normal tay distribution were described using median, 25th quartile and 75th quartile. Between-group differences were assessed using independent samples t-tests for normally distributed variables, and for skewed distribution variables, nonparametric tests with 2 independent samples were used to determine between-group differences in the indicators. The chi-square test was also used to compare between-group differences in dichotomous variables (patient sex, hypertension, history of diabetes mellitus, and smoking history). In the regression analysis, CR2 was regressed as the dependent variable. In univariate regression, each variable was included in the regression analysis separately. variables with *p* < 0.1 were included in the multivariate regression analysis. In multifactorial regression, *p* < 0.05 was regarded as an independent risk factor for changes in CR2.

## Result

Excellent interobserver and intraobserver reliability can be observed in this study. In which, the ICC value of all imaging based parameters were > 0.8 in both interobserver and intraobserver reliability judgement.

No statistically significant differences were observed in preoperative signal change ratio (SCR), canal narrowing ratio (CNR), C2–7 Cobb angle, sagittal vertical axis (SVA), modified K-line interval (mK-line INT), and JOAs between the patients in the anterior and posterior surgery groups (*p* > 0.05, Table 1). Also, at six months postoperatively, there was no significant difference in the improvement of JOAs (Recovery1) and the improvement of HCS (CR1) between the two groups (*p* > 0.05, Table 1). However, compared with the posterior surgery group, the anterior surgery group had significantly better improvement in JOAs (Recovery2) and improvement in HCS (CR2) at two years postoperatively, and the difference was statistically significant (*p* < 0.05, Table 1). Multifactorial logistic regression analysis revealed that posterior surgery and higher preoperative SCR were identified as risk factors for poor HCS improvement at the two-year postoperative period (*p* < 0.05, Table 2).

## Discussion

The progression of ossification of the posterior longitudinal ligament (OPLL) leads to spinal cord compression and diverse clinical symptoms. Cervical spine surgery, including anterior and posterior approaches, offers effective treatment for cervical spondylotic myelopathy caused by OPLL. Different interbody fusion surgeries are employed for anterior cervical spine surgery, while LP and LF are commonly used for posterior approaches. Factors such as K-line, canal narrowing ratio (CNR), and cervical kyphosis influence the choice of surgical access. Severe kyphosis and high CNR favor anterior surgery, while extensive OPLL with long segments tends towards posterior surgery. However, controversy persists regarding the optimal surgical approach for OPLL.

Magnetic resonance imaging (MRI) plays a crucial role in diagnosing cervical spondylotic myelopathy caused by OPLL. It provides clear visualization of spinal cord compression and intramedullary signal changes. Spinal cord compression gradually forms an high cord signals (HCS) on MRI's T2-weighted images (T2WIs). HCS regression reflects spinal cord repair potential and predicts symptom improvement [7, 8, 23]. The severity of HCS can be assessed using the signal change ratio (SCR) between the localized high signal and normal spinal cord signal at the C7-T1 levels. SCR quantitatively analyzes HCS changes after surgery and measures the degree of HCS regression compared to preoperative HCS, serving as an objective imaging index for assessing postoperative symptom improvement [9, 24]. Studies emphasize that the severity of intramedullary HCS is an important prognostic factor for postoperative neurological function. Mild HCS indicates mild neuropathic changes with greater recovery potential, while severe HCS suggests severe changes and limited recovery potential, with higher HCS signal intensity indicating a higher likelihood of irreversible neuronal loss in the spinal cord [4, 7–9, 24–28]. Therefore, postoperative HCS changes play a significant role in predicting neurologic recovery after cervical spine surgery. Evaluating the impact of surgical approach selection on clinical prognosis based on HCS improvement provides a novel perspective for choosing the appropriate surgical approach for OPLL patients.

Existing studies have primarily focused on qualitatively assessing preoperative HCS in OPLL patients and its correlation with postoperative neurological function. However, no study has yet explored the optimal choice of surgical access in OPLL patients combined with HCS. Our study revealed no significant difference in HCS regression at six months postoperatively between the anterior and posterior surgical groups, while the anterior surgery group exhibited significantly better HCS improvement at two years postoperatively (Fig. 3), considering similar baseline patient information. Meanwhile, JOAs obtained preoperatively as well as at 6 months postoperatively showed no significant difference between the anterior and posterior surgery groups (*p* > 0.05, Table 1), and the JOAs at 2 years postoperatively were significantly better in the anterior surgery group than in the posterior surgery group (*p* < 0.05, Table 1). Moreover, there was a significant correlation between improvement in JOAs (Recovery2) and improvement in HCS (CR2) at 2 years postoperatively, which is consistent with previous studies. Thus, this study's objective quantitative imaging analysis of preoperative and postoperative HCS continuum provides valuable guidance for selecting surgical approaches in clinical practice. Compared to the posterior approach, the anterior approach directly relieves spinal cord compression by removing the ossified ligament. In contrast, the posterior approach indirectly decompresses the spinal cord by enlarging the spinal canal, yet the ossified posterior longitudinal ligament remains at the posterior vertebral body margin. This residual ossification poses a risk of damage from cervical spine movement. The anterior surgery group, however, achieves in-situ decompression by removing the posterior longitudinal ligament, maintaining the original anatomical relationship. Although short-term improvements in cervical HCS were comparable, prolonged stimulation by the ossified posterior longitudinal ligament led to significantly worse HCS improvement in the posterior surgery group over time. These findings align with previous clinical studies assessing neurologic function [12, 16, 18, 29].

**Fig. 4:**
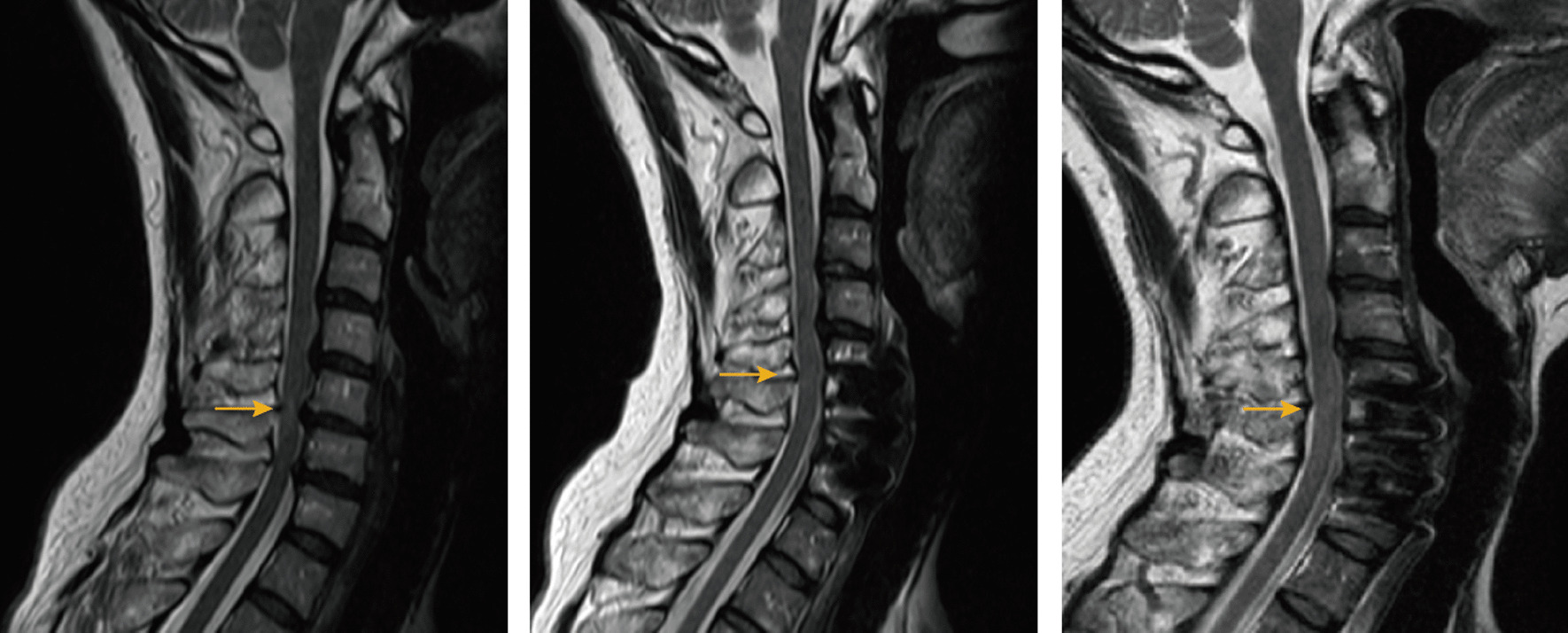
Schematic of HCS changes in the anterior surgery group

**Fig. 5:**
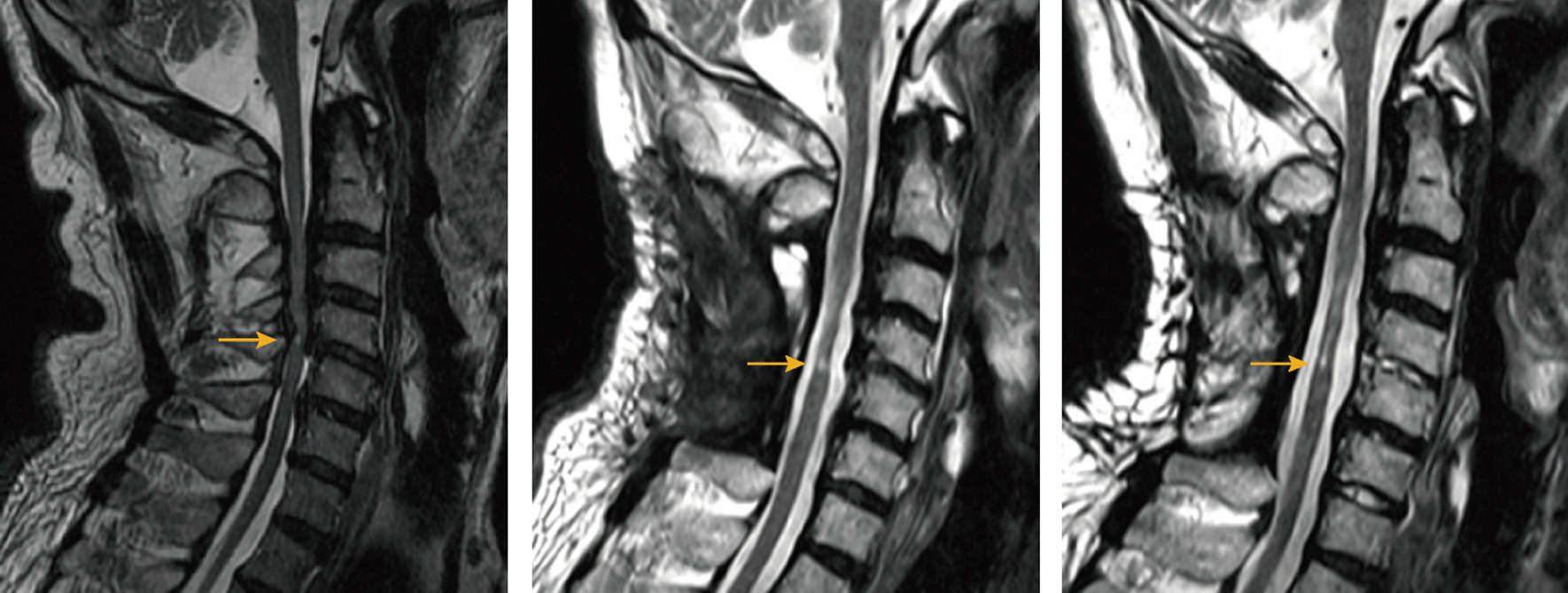
Schematic of HCS changes in the posterior surgery group

Furthermore, patients with cervical spondylotic myelopathy due to OPLL tend to have favorable prognosis with anterior decompression and fusion surgery when CNR is ≥ 60% [12, 14, 18, 29]. However, controversy surrounds the choice of surgical approach for patients with CNR < 60%. In our study, patients with CNR < 60% showed better HCS improvement with anterior surgery compared to posterior surgery. Thus, regardless of CNR, anterior surgery appears more beneficial for patients with cervical spondylotic myelopathy due to OPLL (Figs. 4, 5).

Nevertheless, this study has limitations. It is retrospective with a small sample size, potentially introducing bias in case selection, data measurement, and surgical level influenced by a single operator. For the limited sample size of complication, there were no significant difference in complication rate in patients with anterior and posterior approach cervical operation. The follow-up period was relatively short, and investigating long-term changes in HCS over 5–10 years would be of greater interest. Additionally, this study did not definitively determine the transverse extent and specific location of spinal cord pathological changes caused by HCS, such as central gray or lateral or posterior white columns. Further exploration of the impact of pathologic changes in the anterior horn or posterior pedicle of the spinal cord affected by HCS requires higher-resolution MRI instrumentation.

## Conclusion

For patients with OPLL-induced cervical spondylotic myelopathy and intramedullary high signal, anterior removal of the ossified posterior longitudinal ligament and direct decompression offer a greater potential for regression of intramedullary high signal. At the same time, this anterior surgical strategy improves clinical neurologic function better than indirect decompression in the posterior approach.

From left to right, images are shown preoperatively, six months postoperatively, and two years postoperatively. Anterior surgery group: preoperative SCR = 1.5603; 6 months postoperative SCR = 1.1552; 2 years postoperative SCR = 1.0660.

From left to right, images are shown preoperatively, six months postoperatively, and two years postoperatively. Posterior surgery group: preoperative SCR = 2.1335; six months postoperative SCR = 1.8875; two years postoperative SCR = 2.3153.

## Data Availability

All the data of the manuscript are presented in the paper.

## Abbreviations

BMI: Body mass index
HCS: High cord signal
OPLL: Ossification of the posterior longitudinal ligament
MRI: Magnetic resonance imaging
SCR: The signal change ratio between the localized high signal and normal spinal cord signal at the C7-T1 levels
CR1: The regression of high cord signals at 6 months postoperatively (i.e., CR1 = (Preoperative SCR—SCR at 6 months postoperatively)/ Preoperative SCR)
CR2: The regression of high cord signal at 2 years postoperatively (i.e., CR2 = (Preoperative SCR—SCR at 2 years postoperatively)/ Preoperative SCR)
CNR: Canal narrowing ratio
SVA: Sagittal vertical axis
mK-line INT: Modified K-line interval
JOAs: Japanese Orthopedic Association score
ACDF: Anterior cervical discectomy and fusion
ACCF: Anterior cervical discectomy and fusion
HDF: Hybrid decompression and fusion
LP: Laminoplasty
LF: Laminectomy and fusion
Recovery1: Degree of JOAs recovery at 6 months postoperatively (i.e., Recover1 = (JOAs at 6 months postoperatively—Preoperative JOAs)/ (17- Preoperative JOAs))
Recovery2: Degree of JOAs recovery at 2 years postoperatively (i.e., Recover2 = (JOAs at 2 years postoperatively—Preoperative JOAs)/ (17- Preoperative JOAs))
